# Infectious Respiratory Diseases Decreased during the COVID-19 Pandemic in South Korea

**DOI:** 10.3390/ijerph18116008

**Published:** 2021-06-03

**Authors:** Da Hae Kim, Thi Mai Nguyen, Jin Hee Kim

**Affiliations:** Department of Integrative Bioscience & Biotechnology, Sejong University, 209 Neungdong-ro, Gwangjin-gu, Seoul 05006, Korea; dahae0218@sju.ac.kr (D.H.K.); mainguyen@sju.ac.kr (T.M.N.)

**Keywords:** COVID-19, mitigation measures, infectious respiratory disease, incidence rate, incidence rate ratio

## Abstract

Infectious respiratory diseases are highly contagious and very common, and thus can be considered as one of the leading causes of morbidity and mortality worldwide. We followed up the incidence rates (IRs) of eight infectious respiratory diseases, including chickenpox, measles, pertussis, mumps, invasive pneumococcal disease, scarlet fever, rubella, and meningococcal disease, after COVID-19 mitigation measures were implemented in South Korea, and then compared those with the IRs in the corresponding periods in the previous 3 years. Overall, the IRs of these diseases before and after age- or sex-standardization significantly decreased in the intervention period compared with the pre-intervention periods (*p* < 0.05 for all eight diseases). However, the difference in the IRs of all eight diseases between the IRs before and after age-standardization was significant (*p* < 0.05 for all periods), while it was not significant with regard to sex-standardization. The incidence rate ratios for eight diseases in the pre-intervention period compared with the intervention period ranged from 3.1 to 4.1. These results showed the positive effects of the mitigation measures on preventing the development of respiratory infectious diseases, regardless of age or sex, but we need to consider the age-structure of the population to calculate the effect size. In the future, some of these measures could be applied nationwide to prevent the occurrence or to reduce the transmission during outbreaks of these infections. This study provides evidence for strengthening the infectious disease management policies in South Korea.

## 1. Introduction

Infectious respiratory diseases are highly contagious and very common, and thus can be considered as one of the leading causes of morbidity and mortality worldwide [1]. Because respiratory infections may range from asymptomatic to life-threatening in the case of diseases such as pneumonia and sepsis [2], they are highly threatening to vulnerable populations such as children and the elderly due to their weaker immune systems [3,4]. For this reason, infectious respiratory diseases can potentially overwhelm medical systems and can lead to disruptions of social systems, as we have seen recently in the case of the novel coronavirus [1]. Therefore, to effectively manage these diseases, it is important to diagnose new cases quickly, treat diagnosed patients with appropriate protocols, and finally, to interrupt the public spread of the diseases [1].

As one element of the strategies aimed at controlling infectious diseases, most countries in the world have designated severe infectious respiratory diseases as national notifiable diseases [5]. Prime examples in South Korea include chickenpox (also known as varicella), measles, pertussis, mumps, invasive pneumococcal disease (IPD), scarlet fever, rubella, and meningococcal disease (MD). Viral infections may break out unpredictably at any time and have the potential to become emerging threats to human health and global stability [6]. This is because rapid viral spread from person to person can occur through respiratory droplets produced by sneezing, coughing, and speaking, leading to nationwide epidemics [7]. More importantly, infectious symptoms may worsen, causing serious complications, especially in vulnerable populations. In particular, chickenpox, measles, pertussis, and IPD can induce infections in the lung (i.e., pneumonia) and infection or inflammation in the brain (i.e., encephalitis) [8]. Mumps, scarlet fever, and MD can cause encephalitis, rheumatic fever, and nervous system disorders, respectively [8]. Rubella can cause heart problems, loss of hearing and eyesight, and miscarriage or stillbirth in pregnant women [8]. Fortunately, these diseases and their complications can be effectively prevented through various intervention programs [9]. In South Korea, the National Vaccination Program (NIP) has been implemented to prevent infectious diseases since 1954. The Korean NIP provides free immunization services for chickenpox, measles, pertussis, mumps, IPD, and rubella for children under 12 years of age [10]. The IPD vaccine is also provided for elderly people aged 65 or over. Despite high rates of vaccination coverage [11], these infections have not been completely eliminated [12,13]. For this reason, along with vaccinations, non-pharmaceutical interventions such as hand hygiene, food sanitation, and mask-wearing could be important as well [14].

The 2019 novel coronavirus (COVID-19) pandemic changed the whole world. In South Korea, the first COVID-19 case was diagnosed on 20 January 2020, and a total of 56,359 cases were recorded up until the last day of December 2020 [15]. Facing a dramatic increase in cases, the Korean government implemented a set of measures to protect the nation from COVID-19, including intensified border controls (i.e., obligatory infection testing before being admitted into Korea), picking up hidden cases for isolation, maintaining social distance among persons, and obligatory mask-wearing, and so on [15]. In the absence of vaccines during the early stage of the pandemic, these implementations played an important role in preventing the spread of the virus [16]. Because COVID-19 is itself an infectious respiratory disease, we hypothesized that the COVID-19 mitigation measures could also affect the incidences of the aforementioned respiratory diseases [17]. Because these measures were not widely applied in South Korea before the COVID-19 outbreak [18], it was felt that this pandemic provided an opportunity to investigate their national level impact on common respiratory infections. In the present study, we followed up the incidences of eight infectious respiratory diseases after the initiation of COVID-19 mitigation measures in South Korea, and then we compared them with those before the pandemic. Because the developments in these infectious respiratory diseases were associated with seasonal changes in South Korea [19], we further compared the incidences of infectious respiratory diseases on a monthly basis. In addition, because existing evidence showed that the population structure could influence the onset and recurrence of infectious diseases [20], we also conducted age- and sex-standardization analyses.

## 2. Materials and Methods

### 2.1. Study Design and Target Diseases

This study was an ecological study conducted nationally, representing whole national data in South Korea. The COVID-19 mitigation measures were implemented from 9 March 2020 [21] and data were collected until 31 December 2020. We defined the duration from 9 March to 31 December in 2020 as the intervention period, and the duration from 9 March to 31 December in 2019, 2018, or 2017 as the pre-intervention periods.

Our target infectious respiratory diseases were chickenpox, measles, pertussis, mumps, IPD, scarlet fever, rubella, and MD. These diseases were selected from notifiable diseases that are required to be reported to the Korean government by law among the respiratory infections that are transmitted by droplets or are airborne spread [7].

### 2.2. Calculation of Incidence Rate (IR) and Incidence Rate Ratio (IRR) for the Eight Infectious Respiratory Diseases

The IR was defined as the number of new cases for a target disease divided by the Korean resident registration population (RRP). Because the Korean Statistical Information Service (KOSIS) does not provide the RRP in a specific month, we used only the RRP in a specific year to calculate both the yearly and monthly IRs. Therefore, yearly and monthly IRs were calculated per 100,000 people using the following equation:

$${\mathrm{IR}}_{\mathrm{year}\;(\mathrm{or}\;\mathrm{month})}=\frac{\mathrm{Number}\;\mathrm{of}\;\mathrm{new}\;\mathrm{cases}\;\mathrm{in}\;a\;\mathrm{year}\;(\mathrm{or}\;\mathrm{month})}{\mathrm{RRP}\;\mathrm{in}\;a\;\mathrm{year}}\;\times \;100,000$$

We used the population structure of the Korean RRP in 2005 as a standard population to calculate the age- and sex-standardized IR (AS-IR and SS-IR), according to Statistics Korea’s recommendation [22]. The equation for the AS-IR or the SS-IR was as follows:

$$\mathrm{AS}-\mathrm{IR}\;(\mathrm{or}\;\mathrm{SS}-\mathrm{IR})=\frac{{(\mathrm{IR}}_{\mathrm{year}\;(\mathrm{or}\;\mathrm{month})\;}\mathrm{for}\;a\;\mathrm{target}\;\mathrm{age}\;(\mathrm{or}\;\mathrm{sex})\;\mathrm{group}\;\times \;\mathrm{RRP}\;\mathrm{in}\;a\;\mathrm{year}\;\mathrm{for}\;a\;\mathrm{target}\;\mathrm{age}\;(\mathrm{or}\;\mathrm{sex})\;\mathrm{group}}{\mathrm{RRP}\;\mathrm{in}\;2005}$$

We also calculated the yearly (or monthly) IRR as a ratio of the IR in a pre-intervention year (or month) to the IR in an intervention year (or month) to determine by how much the risk for a disease will be reduced following the intervention. Yearly or monthly IRRs were calculated as follows:

$${\mathrm{IRR}}_{\mathrm{pre}-\mathrm{intervention}\;\mathrm{year}\;(\mathrm{or}\;\mathrm{month})=\;}\frac{\mathrm{IR}\;\mathrm{in}\;a\;\mathrm{pre}-\mathrm{intervention}\;\mathrm{year}\;(\mathrm{or}\;\mathrm{month})}{\mathrm{IR}\;\mathrm{in}\;\mathrm{an}\;\mathrm{intervention}\;\mathrm{year}\;(\mathrm{or}\;\mathrm{month})}$$

Age- and sex-standardized IRRs (AS-IRR and SS-IRR) were calculated as well using the following equation:

$${\mathrm{AS}-\mathrm{IRR}\;(\mathrm{or}\;\mathrm{SS}-\mathrm{IRR})}_{=\;}\frac{\mathrm{AS}-\mathrm{IR}\;(\mathrm{or}\;\mathrm{SS}-\mathrm{IR})\;\mathrm{in}\;a\;\mathrm{pre}-\mathrm{intervention}\;\mathrm{year}\;(\mathrm{or}\;\mathrm{month})}{\mathrm{AS}-\mathrm{IR}\;(\mathrm{or}\;\mathrm{SS}-\mathrm{IR})\;\mathrm{in}\;\mathrm{an}\;\mathrm{intervention}\;\mathrm{year}\;(\mathrm{or}\;\mathrm{month})}$$

### 2.3. Data Collection

The yearly and monthly information for cases in the studied period were collected from the infectious disease portal of the Korea Centers for Disease Control and Prevention Agency (KCDA) [7]. Information about the Korean RRP structures by age and sex in 2020, 2019, 2018, 2017, and 2005 was obtained from the KOSIS [23]. The ethical considerations for the present study were excluded because all the data in our study was anonymous and publicly available.

### 2.4. Data Analyses

To investigate the changes in the incidences of the eight infectious respiratory diseases following the COVID-19 mitigation measures, we compared the yearly IRs of these diseases in the intervention period with those in the pre-intervention period. In addition, considering the seasonal dependency of these diseases, we compared the monthly IRs of these diseases in the intervention period with those in the pre-intervention period. The age- and sex-standardized IRs were compared with the corresponding IRs before the standardization to control for changes in the Korean population structure in the studied periods. Yearly and monthly IRRs, AS-IRRs, and SS-IRRs were also compared with each other to determine the reduction in the risk for a specific disease following the intervention. To further investigate how differently the mitigation measures affected the IRs in different age groups, we calculated and evaluated the differences among the yearly IRs of the eight diseases for each age group before and during the pandemic. All comparisons for population structures, IRs, AS-IRs, SS-IRs, IRRs, AS-IRRs, and SS-IRRs were conducted using the Chi-squared test.

All statistical analyses were performed using the R version 3.6.3 (R Foundation for Statistical Computing, Vienna, Austria).

## 3. Results

### 3.1. Characteristics of Study Population

Table 1 shows the Korean RRP structure by age and sex from 2017 to 2020 and in 2005. Overall, the Korean RRP structure by each age group fluctuated significantly among these years (*p* < 0.05 for all comparisons between two among the studied years) (Table 1). In each year, there were significant differences in the proportions of people in different age groups (*p* < 0.05 for all comparisons between two among the eight age groups). The age groups accounting for the biggest proportions were those in their fifties in 2020 (16.7%), 2019 (16.7%), and 2018 (16.6%), those in their forties in 2017 (16.8%), and those in their thirties in 2005 (18.2%). There was an upward trend in the aged populations and a downward trend in the younger populations. The proportions of males and females changed significantly from 2017 to 2020 (*p* < 0.05 for all comparisons between two among the studied years) (Table 1).

### 3.2. Yearly and Monthly IRs, AS-IRs, and SS-IRs of Eight Infectious Respiratory Diseases in Intervention and Pre-Intervention Periods

Yearly IRs, AS-IRs, and SS-IRs of the eight diseases are shown in Table 2. Among them, chickenpox was the most frequent, followed by mumps and scarlet fever for all years.

Overall, there were significant decreases in the IRs, AS-IRs, and SS-IRs of all eight diseases after the COVID-19 mitigation practices were initiated (*p* < 0.05 for all years). With the exception of rubella, the seven remaining infectious diseases showed similar relations. The IR, AS-IR, and SS-IR of rubella in 2020 were significantly smaller than those in 2019 and 2017. However, we did not find any statistical difference for the IR, the AS-IR, and the SS-IR in relation to 2018 because there was no confirmed case of rubella in 2018.

When we compared the IRs before and after age-standardization, the AS-IRs of chickenpox, mumps, and scarlet fever were significantly increased compared with the IRs before age-standardization of those three diseases (*p* < 0.05 for all three diseases), while there was an inverse relationship for IPD (*p* < 0.05). However, the IRs of rubella and MD did not change after age-standardization. All the IRs for each age group of all eight diseases in the intervention period were significantly smaller than those in the pre-intervention periods (Appendix A, Table A1). For most diseases, the IRs were biggest in the 0–9 age group. For IPD, the IR was big not only in children 0–9 years old, but also in the population that were over 70 years of age. Among these age groups, only the 0–9 group had significantly lower IRs of chickenpox, pertussis, mumps, IPD, and scarlet fever in 2020 than in all the pre-intervention years (*p* < 0.05 for all years). With regard to sex-standardization, we did not find any considerable changes in the IRs of all eight diseases after sex-standardization. For this reason, we did not consider sex-standardization further in our subsequent analyses.

**Figure 1:**
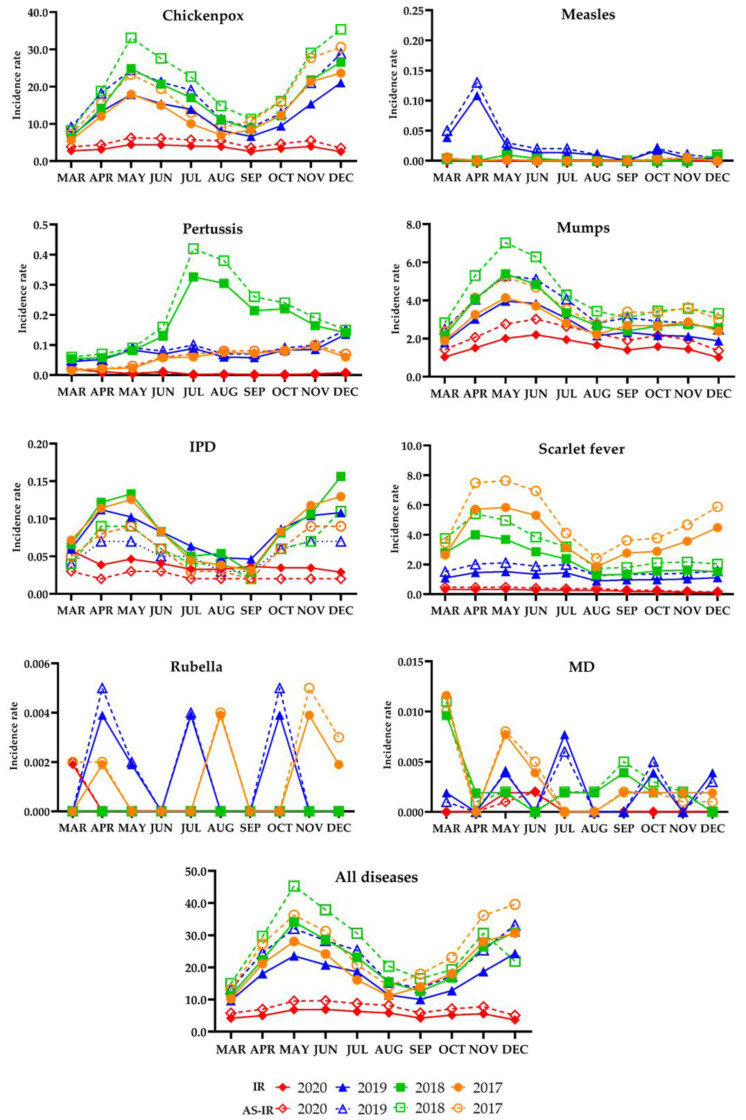
IR and AS-IR of infectious respiratory diseases by year. Units per every 100,000 people.

The monthly IRs and AS-IRs of all eight diseases were significantly lower in the intervention period compared with the pre-intervention period (*p* < 0.05 for all months in the three pre-intervention years) (Figure 1 and Appendix A, Table A2).

A similar pattern was observed in all the monthly IRs and AS-IRs of chickenpox and mumps. Both the IRs and the AS-IRs of chickenpox and mumps increased from March to May regardless of interventions. In particular, the incidences of chickenpox were increasing again in the last three months of all the pre-intervention years with the highest IR or AS-IR in December. Although scarlet fever also showed increasing IRs from March to May in the pre-intervention period, the AS-IRs in 2018 were a little different with a higher AS-IR in April compared with March and May. However, the IRs and the AS-IRs of scarlet fever during the three months of the intervention year were not significantly changed. Overall, the monthly AS-IRs of chickenpox, mumps, and scarlet fever were significantly higher compared with the IRs before standardization (*p* < 0.05 for all).

For pertussis, all the monthly IRs and AS-IRs in the intervention period were significantly smaller than those in the pre-intervention period, except for those in March and April 2017. The AS-IRs in July, August, and September 2018 were significantly higher than the IRs before standardization (*p* < 0.05 for all). The IRs of IPD were reduced in the intervention period compared with the pre-intervention period, from October to December in all three pre-intervention years, from April to July in 2019, and from April to June in 2018 and 2017, while the AS-IRs of IPD were also reduced in the intervention period, from October to December in all three pre-intervention years, in April and May in 2019, and from April to June in 2018 and 2017 (*p* < 0.05 for all). However, the overall monthly AS-IRs of IPD were not significantly different from the corresponding IRs of IPD (*p* > 0.05 for all).

Because there was no confirmed case of measles in the entire intervention period and small numbers of cases for rubella and MD in specific months of the intervention year (one rubella case in March and two MD cases in May and June), the monthly IRs and AS-IRs of both diseases were mostly calculated as zero.

### 3.3. Yearly and Monthly IRRs and AS-IRRs for Eight Infectious Respiratory Diseases in the Intervention and Pre-Intervention Periods

Table 3 presents the yearly IRRs and AS-IRRs for the eight diseases. The yearly IRRs and AS-IRRs for all diseases except measles in all years and rubella in 2018, were significantly greater than one (Table 3). We could not calculate the IRR and the AS-IRR for measles in all years and for rubella in 2018, because there was no confirmed case of measles in 2020 or of rubella in 2018, respectively (Table 2). In general, the risks for pertussis and scarlet fever were dramatically reduced in the intervention period (8.3–25.1 times of reduction in the IRR for pertussis and 4.9–16.0 times of reduction in the IRR for scarlet fever). The smallest reduction in risk in the intervention period was observed for mumps (1.7–2.1 reduction) (Table 3). All the yearly IRRs for pertussis, IPD, rubella, and MD increased after age-standardization, whereas almost all the yearly AS-IRRs for chickenpox, mumps, and scarlet fever were smaller than the corresponding IRRs (Table 3). However, significant differences in the yearly IRRs for chickenpox, mumps, scarlet, pertussis, IPD, rubella, and MD before and after age-standardization were not found.

The monthly IRRs and AS-IRRs for the eight respiratory diseases are shown in Table 4. The monthly IRRs and AS-IRRs for all the diseases in all the studied months ranged from 1.9 to 8.4 and from 1.8 to 7.8, respectively (Table 4). The monthly IRRs and AS-IRRs for chickenpox, mumps, and scarlet fever were all significantly greater than one in all months (*p* < 0.05 for all), indicating significant reductions in the risk for these diseases following the mitigation measures. In particular, the monthly IRRs and AS-IRRs for both chickenpox and scarlet fever in each pre-intervention year reached peaks in December. The IRRs and the AS-IRRs in December for chickenpox were 8.4 and 8.2 in 2019, 10.6 and 10.0 in 2018, and 9.4 and 8.6 in 2017, respectively. Similarly, the IRRs and the AS-IRRs in December for scarlet fever were 9.9 and 9.6 in 2019, 13.5 and 12.7 in 2018, and 40.0 and 36.5 in 2017, respectively. After age-standardization, the monthly IRRs for these three diseases mostly decreased. However, the difference was not significant.

The risk for pertussis from March to December was significantly less in the intervention period compared with the pre-intervention period, except for March and April in 2017 (*p* < 0.05 for all). The monthly IRR for pertussis from July to December increased after age-standardization, with the biggest increase in July 2018 (from 169.0 to 481.6). However, we did not find a significant difference.

There were significant reductions in the risk for IPD from April to June and from October to December in 2020, compared with 2017 and 2018, both before and after age-standardization (2.0–5.4 times of reduction and 2.1–6.3 times of reduction, respectively). In relation to 2019, the IRR for IPD was significantly larger than one in these months and in July as well; however, after age-standardization, this significance was only observed in April, May, and from October to December. We could not calculate the monthly IRRs and AS-IRRs for measles, because no case of measles was reported in all the months of the intervention year. Similarly, we could calculate the monthly IRRs and AS-IRRs for rubella in March only, and for MD only in May and June, because in the intervention period, newly confirmed cases were only reported in these months.

## 4. Discussion

Our study about the eight infectious respiratory diseases in South Korea between 2017 and 2020 showed that chickenpox was the most common, followed by mumps and scarlet fever, whereas the incidences of measles, rubella, and MD were very small. Overall, the prevention of the eight diseases was marked after the mitigation measures were introduced to limit the spread of COVID-19.

Despite national programs to prevent and manage chickenpox, mumps, and scarlet fever, there were outbreaks of these infections in the pre-pandemic period. Indeed, although the vaccination rates for chickenpox and mumps have been increasing since the first implementation of vaccines at the beginning of the 20th century, there was an upward trend in incidences from 2006 to 2019 in South Korea [24,25]. Evidence from a previous study indicated that Suduvax, the most popular vaccine for chickenpox in South Korea, may be insufficiently immunogenic to prevent this disease [26]. For mumps, despite the high efficacy of the vaccine, there are many other factors that are involved in the occurrence of mumps outbreaks [25,27]. Similar to our findings, a study by Huh K et al. [28] also reported that the IRs of chickenpox and mumps after the COVID-19 outbreak were significantly lower than the expected IRs calculated from a regression predictive model. On the other hand, scarlet fever can only be prevented by non-pharmaceutical measures because there is no available vaccine [8]. Therefore, the decreases in incidences of chickenpox, mumps, and scarlet fever during the COVID-19 pandemic suggest a potential benefit of the community mitigation measures on preventing the outbreaks of these common infections.

Measles, rubella, and MD were not prevalent in our study. Indeed, in 2014, South Korea was declared “measles free” by the World Health Organization (WHO) [29]. Despite a small outbreak in 2019 due to “imported” cases, measles has been successfully maintained at the level of elimination [30]. In 2017, the WHO certified South Korea as the first country in the Western Pacific region to eradicate rubella [30]. Although there were great concerns about the under-reported incidence of MD [31], the diagnostic methods used in South Korea were unchanged throughout the studied years [32]. Therefore, despite very small numbers of new cases detected annually, the incidences of measles, rubella, and MD were significantly reduced in the intervention period, suggesting that the COVID-19 mitigation measures may not only sustain, but even strengthen the successes gained in managing these diseases in South Korea.

The monthly incidences of chickenpox, mumps, and scarlet fever in the pre-intervention period of our study showed a consistent increase from April to May, similar to those of two previously reported studies [33,34]. After that, chickenpox decreased from May to September before increasing again in the last three months of the year, but mumps and scarlet fever decreased from May to December. Despite having a much smaller incidence, IPD shared a relatively similar infection trend with chickenpox. The fluctuations in the incidences of these infectious diseases throughout a year are likely to be attributable to seasonal changes in South Korea [19,35]. Previous studies found that chickenpox and IPD viruses do not transmit well under conditions of high ambient temperatures and high humidity, which are representative of summer, lasting from June to August [36,37]. A recent study conducted in Taiwan by Cooper et al. [38] also reported an unintended decline in IPD along with influenza from February to May following the public interventions implemented to prevent the outbreak of COVID-19, including the use of face masks, sanitizer, and social distancing. However, this study did not consider the trends for IPD in the following months, especially in the winter season when the infection is more prevalent. Following up the incidences of IPD from March to December in both the intervention and the pre-intervention periods allowed us to observe not only the natural trend, but also the significant reduction in this infection following the implementation of the nationwide mitigation measures in most seasons of a year. Compared with the pre-intervention period, our study found that the incidences of chickenpox, mumps, and scarlet fever only slightly fluctuated, and remained significantly lower from March to December in the intervention period. This finding provides evidence for developing a plan to prevent or ease seasonal outbreaks of these infections. More interestingly, for pertussis, in the pre-intervention period, the incidence in 2018 was much higher than in 2017 and 2019, especially in July. This is because pertussis is cyclical in nature, with peaks of infection every two to three years in South Korea; over the past decade, pertussis outbreaks were recorded in 2012, 2015, and 2018 [39]. Following this trend, the next outbreak was predicted to occur in 2020 or 2021. Our study found that the incidence of pertussis in the intervention period was very small, which could be due to the simultaneous implementation of COVID-19 mitigation measures in 2020. It may be anticipated that the predicted upcoming pertussis outbreak in 2021 can potentially be controlled if these interventions are still applied nationwide.

Regarding the results obtained from the age- and sex-standardization analyses, significant differences in both the yearly AS-IR and SS-IR between the intervention and the pre-intervention periods and all the IRRs and the AS-IRRs, except for those impossible to compute or calculated as zero because there was no case greater than one, confirmed the robustness of our finding that the community mitigation measures could reduce the development of all eight infectious respiratory diseases. The non-significant differences in the IRs before and after sex-standardization in the intervention and pre-intervention periods indicated similar susceptibilities to infections between males and females. However, after age-standardization, there were significant increases in the IRs of chickenpox, mumps, and scarlet fever but a significant decrease in the IR of IPD in the studied period, suggesting that these infections occur differentially depending on age groups. Our findings are in line with recent evidence showing that the association between the number of new COVID-19 cases and age groups was statistically significant, but this did not occur with gender groups [40]. Our additional analyses by age group provided further information that the 0–9 age group was the most susceptible to chickenpox, pertussis, mumps, scarlet fever, and IPD. Interestingly, the COVID-19 mitigation measures could effectively protect this population against these infections. This is supported by findings from a previous study by Huh et al. [28], which show that following a series of non-pharmaceutical interventions during the COVID-19 outbreak in South Korea, the incidence of chickenpox declined to the largest extent in the 7–17 age group, followed by that in children aged 0–6 years, while mumps showed a marginal reduction in incidences in these two groups, but not in adults [28]. On the other hand, because of the significant differences between the 2020 and 2005 population structures, we also calculated AS-IRs and AS-IRRs using the Korean RRP in 2020 as a standard population. With regard to sex-standardization, we did not find any considerable change after sex-standardization. However, even after age-standardization, the incidences of the eight diseases still significantly decreased in the intervention period, even when the AS-IRs and the AS-IRRs were calculated using the RRP in 2020 (data not shown here).

Because the mask-wearing intervention began on 9 March 2020, we could not include data from 1 to 8 March 2020 in the intervention period. To investigate how the intervention affected the incidences of the eight infectious diseases for the full month of March 2020, we attempted to estimate the incidences under an assumption that the intervention was implemented from 1 March 2020 and compared the estimated values with the real incidences in the pre-intervention years recorded by the KCDA. Because daily numbers of confirmed cases of chickenpox, mumps, and scarlet fever from 1 to 8 March, from 9 to 31 March, and in the full month of March 2020 all followed standard normal distribution (i.e., Gaussian distribution), we estimated the numbers of confirmed cases from 1 to 8 March (eight days) in proportion to the period of March using data from 9 to 31 March (23 days). However, because pertussis and IPD were not normally distributed, we performed a log transformation before calculating the estimation values in the same manner. There were no measles or MD cases, and only one rubella case reported from 1 to 9 March 2020, thus, we could not estimate the numbers of cases for these diseases. Overall, we found that the expected incidences were lower than not only the real incidences in 2020, but also those in the three pre-intervention years (data not shown here). Therefore, we could expect a stronger reduction in the IR if the mask-wearing was initiated from 1 March 2020. However, further research with more sophisticated estimation methods is still needed to confirm this finding.

COVID-19 has been relatively well controlled in South Korea because of the implementation of an aggressive “trace, test, and treat” program, in tandem with strict quarantine protocols, which included mask-wearing, handwashing, and social distancing [41]. Our hypothesis that the COVID-19 mitigation measures may contribute to the prevention of not only COVID-19, but also other infectious respiratory diseases was supported by evidence from the United States, Australia, Chile, South Africa, and Sweden [42,43,44]. Because all of these interventions were applied simultaneously, it is very difficult to specify the effect of any particular intervention separately. Nevertheless, there was strong evidence for the superior benefits of mask-wearing over other community mitigation measures on the development of the infectious respiratory diseases in the context of South Korea. First, while the Korean government relaxed several interventions such as social distancing or border controls in specific periods amid the pandemic due to socio-economic benefits [20], mask-wearing was continuously mandated from the beginning of the pandemic because it could be implemented at a minimum cost without dramatically disrupting social practices [45]. Second, considering the cost-effectiveness of mask-wearing, the Korean government banned the export of masks abroad and supported mask production companies to increase the speed of the entire manufacturing process, from sourcing to inventory management, to completely control the production and supply of masks at the national level [46]. These actions by the government resulted in a very high proportion (94%) of Koreans wearing a face mask when in public, which was rated as the best among 28 surveyed countries [41]. Third, droplets containing the viruses that result in the eight target diseases can spread more than two meters [47]. Because the distance for social distancing implemented in South Korea was two meters, infection could still occur by contact with contaminated droplets or aerosols. Interestingly, when the level of social distancing was reduced from May to August [48], the incidences of the eight infections remained low. One possible explanation for this is that mask-wearing, which could greatly reduce the risk for respiratory infections [49], was kept mandated.

Our research has several limitations. The first, which is the biggest limitation, may be related to the reliability and validity of the dataset used in our analyses. Specifically, we were unable to control for variables which could affect the development of the eight diseases due to unavailable individual information. For example, the risk for mumps may increase in environments with a high population density [50], and people with chronic respiratory disease could be more susceptible to IPD [51]. Besides, confirmed cases of the eight target diseases that entered from abroad were not covered in the database. However, as national notifiable diseases, the domestic cases were mandated to be recorded in the KCDA portal on a daily basis by the Korean government. This indicates that the KCDA database could be considered as a reliable and valid data source for studying national notifiable diseases in South Korea. In addition, the nationwide data updated daily allowed us to conveniently and quickly follow up the incidences of the eight target diseases at a national level in both the pandemic and the pre-pandemic periods, which is impossible in studies collecting raw data. More interestingly, because the KCDA systematically provided numbers of cases by age group, sex, and recorded date, we conducted the analyses by age and sex to determine the difference in the effects among age or sex groups because of temporal changes in the Korean population structure, and further considered several seasonal factors due to the different features of the target diseases by season. In a nutshell, the advantages of using national data provided by the KCDA outweighed the disadvantages with regard to the limited information. Second, because of a lack of monthly RRP information, there might be a small bias in the calculations of the monthly values. Third, our study only focused on the duration from March to December, excepting for January and February, when the weather is usually cold and dry in South Korea [35]. Finally, we could not use data from 1 to 8 March in 2020 because this was not part of the intervention period. However, even though these limitations may produce some random errors resulting in a null hypothesis, our results showed statistically significant and protective effects of the COVID-19 mitigation measures on the development of eight infectious respiratory diseases.

## 5. Conclusions

In this study, we found that the incidences of all eight infectious respiratory diseases, including chickenpox, measles, pertussis, mumps, IPD, scarlet fever, rubella, and MD, significantly decreased following the implementation of the COVID-19 community mitigation measures to prevent the spread of COVID-19 in South Korea. Everyone, regardless of age or sex, benefited from the protective effect of these measures. Because of the potential to interfere with the natural circulation of these infectious diseases, the COVID-19 interventions can be considered as effective interventions for preventing or easing seasonal outbreaks of several prevalent infections such as chickenpox, mumps, and scarlet fever. Our study provides evidence for strengthening the infectious disease management policies in South Korea.

## Figures and Tables

**Table 1:** Korean RRP structure by age and sex from 2017 to 2020 and in 2005.

Variables	2020	2019	2018	2017	2005
Korean RRP, N (%)	51,829,023 (100)	51,849,861 (100)	51,826,059 (100)	51,778,544 (100)	48,683,040 (100)
Age group, N (%)					
0–9	3,970,070 (7.7)	4,166,914 (8.0)	4,303,062 (8.3)	4,435,198 (8.6)	5,829,053 (12.0)
10–19	4,793,336 (9.2)	4,959,010 (9.6)	5,131,153 (9.9)	5,304,425 (10.2)	6,670,033 (13.7)
20–29	6,806,153 (13.1)	6,810,356 (13.1)	6,823,973 (13.2)	6,810,967 (13.2)	7,697,455 (15.8)
30–39	6,873,117 (13.3)	7,071,024 (13.6)	7,270,143 (14.0)	7,368,649 (14.2)	8,859,246 (18.2)
40–49	8,294,787 (16.0)	8,383,230 (16.2)	8,488,587 (16.4)	8,702,752 (16.8)	8,325,045 (17.1)
50–59	8,645,014 (16.7)	8,667,377 (16.7)	8,615,884(16.6)	8,490,204 (16.4)	5,079,574 (10.4)
60–69	6,744,506 (13.0)	6,310,651 (12.2)	5,949,639 (11.5)	5,657,264 (10.9)	3,674,784 (7.5)
70+	5,702,040 (11.0)	5,481,299 (10.6)	5,243,618 (10.1)	5,009,085 (9.7)	2,547,850 (5.2)
Sex group, N (%)					
Male	25,841,029 (49.9)	25,864,816 (49.9)	25,866,129 (49.9)	25,855,919 (49.9)	24,409,659 (50.1)
Female	25,987,994 (50.1)	25,985,045 (50.1)	25,959,930 (50.1)	25,922,625 (50.1)	24,273,381 (49.9)

RRP = resident registration population; N = number of persons.

**Table 2:** Yearly IRs, AS-IRs, and SS-IRs of the eight infectious diseases in the intervention and pre-intervention periods.

Category	Year	Chickenpox	Measles	Pertussis	Mumps	IPD	Scarlet Fever	Rubella	MD	All Diseases
No. of cases	2020	18,165	0	35	8139	197	1239	1	2	27,778
	2019	66,284	120	393	13,664	422	6119	7	11	87,020
	2018	84,537	12	877	17,002	456	11,932	0	13	114,829
	2017	69,010	6	290	14,805	434	19,753	7	16	104,321
IR 1	2020 (Ref.)	35.05	0	0.07	15.70	0.38	2.39	0.002	0.004	53.60
	2019	127.84 *	0.23*	0.76 *	26.35 *	0.81 *	11.80 *	0.01 *	0.02 *	167.83 *
	2018	163.12 *	0.02*	1.69 *	32.81 *	0.88 *	23.02 *	0	0.03 *	221.57 *
	2017	133.28 *	0.01*	0.56 *	28.59 *	0.84 *	38.15 *	0.01 *	0.03 *	201.48 *
AS-IR 1	2020 (Ref.)	52.56 †	0	0.07	22.92 †	0.24 †	3.66 †	0.002	0.003	79.47 †
	2019	186.38 *,†	0.88 *,†	0.88 *,†	37.43 *,†	0.57 *,†	17.48 *,†	0.02 *	0.02 *	243.08 *,†
	2018	224.26 *,†	2.10 *	2.10 *,†	44.10 *,†	0.67 *,†	32.04 *,†	0	0.03 *	303.21 *,†
	2017	183.32 *,†	0.64 *	0.64 *	38.38 *,†	0.62 *,†	53.15 *,†	0.02 *	0.03 *	276.18 *,†
SS-IR 1	2020 (Ref.)	35.05	0	0.07	15.71	0.38	2.39	0.002	0.004	53.61
	2019	127.87 *	0.23 *	0.76 *	26.37 *	0.82 *	11.81 *	0.01 *	0.02 *	167.89 *
	2018	163.15 *	0.02 *	1.69 *	32.82 *	0.88 *	23.04 *	0	0.03 *	221.64 *
	2017	133.31 *	0.01 *	0.56 *	28.61 *	0.84 *	38.17 *	0.01 *	0.03 *	201.54 *

IR = incidence rate; AS-IR = age-standardized incidence rate; SS-IR = sex-standardized incidence rate; ^1^ Unit, per 100,000 people; IPD = invasive pneumococcal disease; MD = meningococcal disease; Ref. = reference population; *, *p* < 0.05 for comparisons between the pre-intervention period and the intervention period; ^†^, *p* < 0.05 for comparisons between the IRs and AS-IRs.

**Table 3:** Yearly IRRs and AS-IRRs for eight infectious respiratory diseases.

Category	Year	Chickenpox	Measles	Pertussis	Mumps	IPD	Scarlet Fever	Rubella	MD	All Diseases
IRR	2019	3.6 *	-	11.2 *	1.7 *	2.1 *	4.9 *	7.0 *	5.5 *	3.1 *
	2018	4.7 *	-	25.1 *	2.1 *	2.3 *	9.6 *	-	6.5 *	4.1 *
	2017	3.8 *	-	8.3 *	1.8 *	2.2 *	16.0 *	7.0 *	8.0 *	3.8 *
AS-IRR	2019	3.6 *	-	12.5 *	1.6 *	2.3 *	4.8 *	8.2 *	6.4 *	3.1 *
	2018	4.3 *	-	30.0 *	1.9 *	2.7 *	8.8 *	-	9.1 *	3.8 *
	2017	3.5 *	-	9.1 *	1.7 *	2.6 *	14.6 *	8.3 *	9.8 *	3.5 *

IRR = incidence rate ratio; AS-IRR = age-standardized incidence rate ratio; IPD = invasive pneumococcal disease; MD = meningococcal disease; - = impossible to calculate; *, *p* < 0.05 for comparisons between the rate ratio values and 1. IR in 2020 was used as the denominator for all calculations.

**Table 4:** Monthly IRRs and AS-IRRs for eight infectious respiratory diseases.

Category	Year	Month	Chickenpox	Measles	Pertussis	Mumps	IPD	Scarlet Fever	Rubella	MD	All Diseases
IRR	2019	Mar	2.4 *	-	2.3 *	1.7 *	1.0	3.4 *	0	-	2.3 *
		Apr	4.2 *	-	4.5 *	2.0 *	2.9 *	4.7 *	-	-	3.6 *
		May	4.0 *	-	21.5 *	2.0 *	2.2 *	4.6 *	-	2.0	3.4 *
		Jun	3.5 *	-	6.0 *	1.7 *	2.0 *	4.8 *	-	0	3.0 *
		Jul	3.4 *	-	46.0 *	1.6 *	1.9 *	5.4 *	-	-	3.0 *
		Aug	2.1 *	-	15.5 *	1.3 *	1.4	3.4 *	-	-	2.0 *
		Sep	2.6 *	-	30.0 *	1.7 *	1.3	5.0 *	-	-	2.4 *
		Oct	2.8 *	-	43.0 *	1.4 *	2.5 *	5.5 *	-	-	2.5 *
		Nov	3.9 *	-	22.0 *	1.5 *	3.0 *	8.0 *	-	-	3.4 *
		Dec	8.4 *	-	17.5 *	1.9 *	3.7 *	9.9 *	-	-	6.6 *
	2018	Mar	2.3 *	-	2.8 *	2.1 *	1.1	8.7 *	0	-	2.7 *
		Apr	4.5 *	-	5.0 *	2.7 *	3.2 *	12.9 *	-	-	4.5 *
		May	5.6 *	-	21.0 *	2.7 *	2.9 *	11.1 *	-	1.0	5.0 *
		Jun	4.7 *	-	11.2 *	2.2 *	2.0 *	10.3 *	-	0	4.1 *
		Jul	4.2 *	-	169.0 *	1.7 *	1.5	9.0 *	-	-	3.7 *
		Aug	2.9 *	-	79.0 *	1.6 *	1.6	4.8 *	-	-	2.7 *
		Sep	3.3 *	-	111.0 *	1.7 *	0.8	7.0 *	-	-	3.0 *
		Oct	3.6 *	-	114.0 *	1.7 *	2.3 *	8.8 *	-	-	3.2 *
		Nov	5.5 *	-	42.5 *	1.9 *	3.1 *	12.8 *	-	-	4.7 *
		Dec	10.6 *	-	18.3 *	2.5 *	5.4 *	13.5 *	-	-	8.4 *
	2017	Mar	2.0 *	-	0.8	1.8 *	1.2	8.2 *	1.0	-	2.4 *
		Apr	3.8 *	-	1.8	2.2 *	3.0 *	18.4 *	-	-	4.2 *
		May	4.0 *	-	6.0 *	2.1 *	2.7 *	17.5 *	-	4.0	4.1 *
		Jun	3.4 *	-	5.0 *	1.7 *	2.0 *	19.1 *	-	2.0	3.5 *
		Jul	2.3 *	-	31.0 *	1.4 *	1.4	11.9 *	-	-	2.6 *
		Aug	1.8 *	-	19.5 *	1.3 *	1.2	6.9 *	-	-	1.9 *
		Sep	3.2 *	-	37.0 *	1.9 *	0.8	14.5 *	-	-	3.3 *
		Oct	3.7 *	-	42.0 *	1.7 *	2.4 *	16.2 *	-	-	3.5 *
		Nov	5.4 *	-	24.5 *	2.0 *	3.4 *	28.0 *	-	-	5.0 *
		Dec	9.4 *	-	7.8 *	2.4 *	4.5 *	40.0 *	-	-	8.4 *
AS-IRR	2019	Mar	2.4 *	-	2.5 *	1.7 *	1.1	3.3 *	0	-	2.3 *
		Apr	4.1 *	-	3.6 *	2.0 *	3.2 *	4.5 *	-	-	3.5 *
		May	3.9 *	-	15.4 *	1.9 *	2.5 *	4.4 *	-	3.9	3.4 *
		Jun	3.4 *	-	6.1 *	1.7 *	1.9	4.7 *	-	0	2.9 *
		Jul	3.3 *	-	117.8 *	1.5 *	2.0	5.3 *	-	-	2.9*
		Aug	2.1 *	-	37.7 *	1.2 *	1.7	3.2 *	-	-	1.9 *
		Sep	2.5 *	-	76.8 *	1.6 *	1.2	4.9 *	-	-	2.3 *
		Oct	2.7 *	-	101.0 *	1.3 *	2.9 *	5.3 *	-	-	2.4 *
		Nov	3.8 *	-	31.7 *	1.4 *	4.0 *	7.7 *	-	-	3.3 *
		Dec	8.2 *	-	20.8 *	1.8 *	4.2 *	9.6 *	-	-	6.5 *
	2018	Mar	2.1 *	-	3.0 *	2.0 *	1.4	8.2 *	0	-	2.6 *
		Apr	4.3 *	-	5.3 *	2.6 *	4.0 *	12.0 *	-	-	4.3 *
		May	5.3 *	-	16.3 *	2.6 *	3.3 *	10.4 *	-	1.9	4.8 *
		Jun	4.5 *	-	12.2 *	2.1 *	2.1 *	9.5 *	-	0	4.0 *
		Jul	4.0 *	-	481.6 *	1.6 *	1.6	8.5 *	-	-	3.5 *
		Aug	2.7 *	-	217.3 *	1.5 *	1.8	4.5 *	-	-	2.5 *
		Sep	3.1 *	-	300.5 *	1.6 *	1.0	6.6 *	-	-	2.8 *
		Oct	3.4 *	-	283.1 *	1.6 *	2.7 *	8.3 *	-	-	2.7 *
		Nov	5.2 *	-	61.3 *	1.8 *	4.3 *	11.9 *	-	-	4.0 *
		Dec	10.0 *	-	21.3 *	2.4 *	6.3 *	12.7 *	-	-	4.3 *
	2017	Mar	1.9 *	-	0.8	1.7 *	1.4	7.5 *	0.8	-	2.3 *
		Apr	3.5 *	-	1.6	2.0 *	3.8 *	16.6 *	-	-	3.9 *
		May	3.7 *	-	4.8 *	1.9 *	3.4 *	16.0 *	-	7.2	3.8 *
		Jun	3.2 *	-	4.9 *	1.6 *	2.1 *	17.2 *	-	2.4	3.2 *
		Jul	2.3 *	-	77.0 *	1.3 *	1.2	11.0 *	-	-	2.4 *
		Aug	1.6 *	-	47.3 *	1.2 *	1.4	6.3 *	-	-	1.8 *
		Sep	3.0 *	-	88.7 *	1.8 *	0.9	13.2 *	-	-	3.1 *
		Oct	3.4 *	-	94.5 *	1.5 *	2.7 *	14.9 *	-	-	3.2 *
		Nov	5.0 *	-	33.4 *	1.8 *	5.0 *	25.5 *	-	-	4.7 *
		Dec	8.6 *	-	9.4 *	2.2 *	5.2 *	36.5 *	-	-	7.8 *

IRR = incidence rate ratio; AS-IRR = age-standardized incidence rate ratio; IPD = invasive pneumococcal disease; MD = meningococcal disease; - = impossible to calculate; *, *p* < 0.05 for comparisons between the rate ratio values and 1. IR in 2020 was used as the denominator for all calculations.

## Appendix A

**Table A1:** IRs of eight infectious respiratory diseases in intervention and pre-intervention periods by each age group.

Year	Age Group	Chickenpox	Measles	Pertussis	Mumps	IPD	Scarlet Fever	Rubella	MD	All Diseases
2020 (Ref.)	0–9	328.88	0	0.33	132.77	0.30	26.68	0	0	488.95
	10–19	66.58	0	0.04	29.76	0.02	2.08	0	0	98.48
	20–29	10.60	0	0.01	5.64	0	0.26	0	0	16.52
	30–39	6.49	0	0.03	5.07	0.07	0.52	0	0	12.19
	40–49	3.37	0	0.02	3.66	0.11	0.14	0.01	0.01	7.33
	50–59	2.37	0	0	2.51	0.35	0.02	0	0.01	5.26
	60–69	1.64	0	0.03	1.51	0.57	0.06	0	0	3.80
	70+	1.71	0	0.23	1.11	1.79	0.07	0	0	4.91
2019	0–9	1152.24 *	0.58*	3.89 *	223.11 *	0.96 *	138.47 *	0	0	1519.25 *
	10–19	296.05 *	0.12*	1.75 *	50.03 *	0.10	4.64 *	0	0.04	352.73 *
	20–29	23.42 *	0.90*	0.09	7.97 *	0.04	0.38	0	0.06 *	32.86 *
	30–39	13.86 *	0.28*	0.11	6.86 *	0.13	0.75	0.07 *	0	22.06 *
	40–49	6.30 *	0.08*	0.13 *	4.81 *	0.29 *	0.29 *	0.02	0	11.92 *
	50–59	2.75	0.02	0.17 *	3.12 *	0.66 *	0.07	0	0.02	6.81 *
	60–69	2.19	0	0.30 *	1.71	1.58 *	0.05	0	0.05	5.88 *
	70+	2.03	0	1.55 *	1.40	3.36 *	0.13	0	0	8.47 *
2018	0–9	1452.40 *	0.09*	11.07 *	266.37 *	0.88 *	251.62 *	0	0.05	1982.48 *
	10–19	306.99 *	0.06	3.85 *	57.35 *	0.17 *	11.61 *	0	0.06	380.08 *
	20–29	24.02 *	0.01	0.07	8.74 *	0.10 *	0.41	0	0.06 *	33.42 *
	30–39	14.94 *	0.01	0.19 *	7.86 *	0.28 *	0.87 *	0	0.03	24.18 *
	40–49	6.71 *	0.02	0.20 *	5.29 *	0.33 *	0.33 *	0	0	12.88 *
	50–59	3.10 *	0.01	0.28 *	3.49 *	0.75 *	0.09	0	0.01	7.74 *
	60–69	2.47	0	0.58 *	2.46 *	2.05 *	0.27 *	0	0	7.83 *
	70+	2.24	0	1.78 *	1.54	3.41 *	0.24 *	0	0.02	9.22 *
2017	0–9	1233.41 *	0.07	2.77 *	226.15 *	0.99 *	425.35 *	0.07	0.02	1888.82 *
	10–19	210.48*	0.02	1.19 *	53.84 *	0.09	13.71 *	0.02	0.08 *	279.43 *
	20–29	20.31*	0.01	0.06	8.60 *	0	0.35	0	0.07 *	29.41 *
	30–39	11.87*	0	0.14 *	7.25 *	0.23 *	1.00 *	0.01	0	20.51 *
	40–49	5.42*	0.01	0.14 *	4.22 *	0.31 *	0.38 *	0.02	0	10.50 *
	50–59	2.34	0	0.16 *	3.04	0.91 *	0.14 *	0	0.02	6.62 *
	60–69	1.96	0	0.32 *	1.91	1.59 *	0.12	0	0.05	5.96 *
	70+	2.02	0	0.92 *	1.32	3.47 *	0.22	0	0.02	7.97 *

Unit, per 100,000 people; IPD = invasive pneumococcal disease; MD = meningococcal disease; Ref. = reference population; *, *p* < 0.05 for comparisons between pre-intervention period and intervention period.

**Table A2:** Monthly IRs and AS-IRs of eight infectious respiratory diseases in intervention and pre-intervention periods.

Category	Year	Month	Chickenpox	Measles	Pertussis	Mumps	IPD	Scarlet Fever	Rubella	MD	All Diseases
IR	2020	Mar	2.76	0	0.02	1.04	0.06	0.32	0.002	0	4.19
		Apr	3.13	0	0.01	1.50	0.04	0.31	0	0	5.00
		May	4.44	0	0.004	2.01	0.05	0.33	0	0.002	6.83
		Jun	4.37	0	0.01	2.20	0.04	0.28	0	0.002	6.90
		Jul	4.05	0	0.002	1.94	0.03	0.26	0	0	6.28
		Aug	3.86	0	0.004	1.66	0.03	0.27	0	0	5.82
		Sep	2.59	0	0.002	1.40	0.04	0.19	0	0	4.22
		Oct	3.37	0	0.002	1.58	0.03	0.18	0	0	5.16
		Nov	3.96	0	0.004	1.44	0.03	0.13	0	0	5.57
		Dec	2.51	0	0.008	1.01	0.03	0.11	0	0	3.66
	2019	Mar	6.67 *	0.04 *	0.04 *	1.79 *	0.06	1.09 *	0	0.002	9.70 *
		Apr	13.29 *	0.11 *	0.05 *	3.02 *	0.11 *	1.45 *	0.004	0	18.03 *
		May	17.85 *	0.02 *	0.08 *	3.97 *	0.10 *	1.52 *	0.002	0.004	23.55 *
		Jun	15.43 *	0.01 *	0.07 *	3.83 *	0.08 *	1.35 *	0	0	20.77 *
		Jul	13.92 *	0.01 *	0.09 *	3.07 *	0.06 *	1.43 *	0.004	0.008 *	18.59 *
		Aug	8.22 *	0.01 *	0.06 *	2.14 *	0.05	0.89 *	0	0	11.38 *
		Sep	6.63 *	0	0.06 *	2.32 *	0.05	0.96 *	0	0	10.02 *
		Oct	9.43 *	0.02 *	0.08 *	2.17 *	0.09 *	0.97 *	0.004	0.004	12.77 *
		Nov	15.34 *	0.004	0.08 *	2.11 *	0.10 *	1.02 *	0	0	18.67 *
		Dec	21.06 *	0.004	0.14 *	1.87 *	0.11 *	1.11 *	0	0.004	24.30 *
	2018	Mar	6.25 *	0.002	0.05 *	2.16 *	0.07	2.78 *	0	0.01 *	11.33 *
		Apr	14.13 *	0	0.06 *	4.05 *	0.12 *	4.00 *	0	0.002	22.37 *
		May	24.84 *	0.01 *	0.08 *	5.39 *	0.13 *	3.69 *	0	0.002	34.15 *
		Jun	20.68 *	0.004	0.13 *	4.84 *	0.08 *	2.85 *	0	0	28.59 *
		Jul	17.02 *	0	0.33 *	3.34 *	0.05	2.37 *	0	0.002	23.11 *
		Aug	11.13 *	0	0.30 *	2.67 *	0.05	1.29 *	0	0.002	15.45 *
		Sep	8.57 *	0	0.21 *	2.40 *	0.03	1.34 *	0	0.004	12.55 *
		Oct	12.15 *	0	0.22 *	2.65 *	0.08 *	1.56 *	0	0.002	16.67 *
		Nov	21.80 *	0	0.16 *	2.74 *	0.11 *	1.62 *	0	0.002	26.43 *
		Dec	26.54 *	0.008 *	0.14 *	2.56 *	0.16 *	1.51 *	0	0	30.92 *
	2017	Mar	5.62 *	0.004	0.02	1.88 *	0.07	2.63 *	0.01	0.01 *	10.24 *
		Apr	12.03 *	0	0.02	3.25 *	0.11 *	5.71 *	0.002	0	21.13 *
		May	17.94 *	0.002	0.02 *	4.13 *	0.13 *	5.82 *	0	0.01	28.05 *
		Jun	14.99 *	0	0.06 *	3.71 *	0.08 *	5.30 *	0	0.004	24.15 *
		Jul	10.05 *	0	0.06 *	2.81 *	0.04	3.14 *	0	0	16.11 *
		Aug	6.95 *	0	0.08 *	2.20 *	0.04	1.85 *	0.004	0	11.12 *
		Sep	8.37 *	0	0.07 *	2.68 *	0.03	2.76 *	0	0.002	13.92 *
		Oct	12.31 *	0.002	0.08 *	2.67 *	0.08 *	2.88 *	0	0.002	18.03 *
		Nov	21.37 *	0.004	0.09 *	2.86 *	0.12 *	3.57 *	0.004	0.002	28.02 *
		Dec	23.64 *	0	0.06 *	2.39 *	0.13 *	4.48 *	0.002	0.002	30.70 *
AS-IR	2020	Mar	3.85 †	0	0.02	1.42 †	0.03	0.46 †	0.002	0	5.79 †
		Apr	4.40 †	0	0.01	2.06 †	0.02	0.45 †	0	0	6.94 †
		May	6.25 †	0	0.006	2.76 †	0.03	0.48 †	0	0.001	9.52 †
		Jun	6.14 †	0	0.01	3.02 †	0.03	0.40 †	0	0.002	9.60 †
		Jul	5.72 †	0	0.001	2.64 †	0.02	0.37 †	0	0	8.75 †
		Aug	5.46 †	0	0.002	2.27 †	0.02	0.38 †	0	0	8.13 †
		Sep	3.61 †	0	0.001	1.91 †	0.02	0.27 †	0	0	5.81 †
		Oct	4.67 †	0	0.001	2.16 †	0.02	0.25 †	0	0	7.11 †
		Nov	5.55 †	0	0.003	1.96 †	0.02	0.18 †	0	0	7.71 †
		Dec	3.53 †	0	0.007	1.36 †	0.02	0.16 †	0	0	5.08 †
	2019	Mar	9.13 *,†	0.05 *	0.05 *	2.48 *,†	0.04	1.52 *,†	0	0.001	13.26 *,†
		Apr	18.20 *,†	0.13 *	0.05 *	4.06 *,†	0.07 *	2.02 *,†	0.005	0	24.53 *,†
		May	24.48 *,†	0.03 *	0.09 *	5.30 *,†	0.07 *	2.12 *,†	0.002	0.004	32.09 *,†
		Jun	21.17 *,†	0.02 *	0.08 *	5.11 *,†	0.05	1.88 *,†	0	0	28.31 *,†
		Jul	19.09 *,†	0.02 *	0.10 *	4.06 *,†	0.05	1.99 *,†	0.004	0.006	25.31 *,†
		Aug	11.21 *,†	0.01 *	0.07 *	2.81 *,†	0.03	1.24 *,†	0	0	15.37 *,†
		Sep	9.01 *,†	0	0.07 *	3.09 *,†	0.03	1.34 *,†	0	0	13.53 *,†
		Oct	12.83 *,†	0.02 *	0.09 *	2.91 *,†	0.06 *	1.35 *,†	0.005	0.005	17.27 *,†
		Nov	21.00 *,†	0.01	0.10 *	2.82 *,†	0.07 *	1.42 *,†	0	0	25.41 *,†
		Dec	28.88 *,†	0.005	0.15 *	2.49 *,†	0.07 *	1.55 *,†	0	0.003	33.15 *,†
	2018	Mar	8.26 *,†	0.003	0.06 *	2.82 *,†	0.04	3.76 *,†	0	0.01 *	14.96 *,†
		Apr	18.79 *,†	0	0.07 *	5.30 *,†	0.09 *	5.40 *,†	0	0.001	29.66 *,†
		May	33.12 *,†	0.01 *	0.09 *	7.03 *,†	0.09 *	4.97 *,†	0	0.002	45.32 *,†
		Jun	27.56 *,†	0.003	0.16 *	6.29 *,†	0.06 *	3.84 *,†	0	0	37.91 *,†
		Jul	22.68 *,†	0	0.42 *,†	4.29 *,†	0.04	3.19 *,†	0	0.002	30.62 *,†
		Aug	14.78 *,†	0	0.38 *,†	3.44 *,†	0.04	1.73 *,†	0	0.002	20.36 *,†
		Sep	11.34 *,†	0	0.26 *,†	3.09 *,†	0.02	1.80 *,†	0	0.005	16.52 *,†
		Oct	16.08 *,†	0	0.24 *	3.45 *,†	0.06 *	2.10 *,†	0	0.003	19.29 *,†
		Nov	29.05 *,†	0	0.19 *	3.57 *,†	0.07 *	2.18 *,†	0	0.002	30.58 *,†
		Dec	35.38 *,†	0.01 *	0.15 *	3.32 *,†	0.11 *	2.04 *,†	0	0	21.94 *,†
	2017	Mar	7.25 *,†	0.005	0.02	2.36 *,†	0.05	3.45 *,†	0.002	0.01 *	13.14 *,†
		Apr	15.58 *,†	0	0.02	4.13 *,†	0.08 *	7.48 *,†	0.002	0	27.30 *,†
		May	23.23 *,†	0.002	0.03 *	5.24 *,†	0.09 *	7.63 *,†	0	0.008	36.23 *,†
		Jun	19.42 *,†	0	0.06 *	4.69 *,†	0.06 *	6.94 *,†	0	0.005	31.17 *,†
		Jul	13.00 *,†	0	0.07 *	3.53 *,†	0.03	4.11 *,†	0	0	20.75 *,†
		Aug	8.98 *,†	0	0.08 *	2.76 *,†	0.03	2.42 *,†	0.004	0	14.27 *,†
		Sep	10.78 *,†	0	0.08 *	3.40 *,†	0.02	3.62 *,†	0	0.002	17.90 *,†
		Oct	15.83 *,†	0.003	0.08 *	3.35 *,†	0.06 *	3.77 *,†	0	0.002	23.10 *,†
		Nov	27.69 *,†	0.005	0.10 *	3.62 *,†	0.09 *	4.67 *,†	0.005	0.001	36.18 *,†
		Dec	30.59 *,†	0	0.07 *	3.00 *,†	0.09 *	5.87 *,†	0.003	0.001	39.62 *,†

IR = incidence rate; AS-IR = age-standardized incidence rate; Unit, per every 100,000 people; IPD = invasive pneumococcal disease; MD = meningococcal disease; Ref. = reference population *, *p* < 0.05 for comparisons between pre-intervention period and intervention period (reference year is 2020); †, *p* < 0.05 for comparisons between IRs and AS-IRs.
